# *Staphylococcus aureus* mutants resistant to the feed-additive monensin show increased virulence and altered purine metabolism

**DOI:** 10.1128/mbio.03155-23

**Published:** 2024-01-12

**Authors:** Omar M. Warsi, Lina M. Upterworth, Annika Breidenstein, Ulrika Lustig, Kasper Mikkelsen, Tamás Nagy, Dávid Szatmari, Hanne Ingmer, Dan I. Andersson

**Affiliations:** 1 Department of Medical Biochemistry and Microbiology, Uppsala University, Uppsala, Sweden; 2 Department of Evolutionary Ecology and Genetics, Zoological Institute, Kiel University, Kiel, Germany; 3 Department of Medical Chemistry and Biophysics, Umeå University, Umeå, Sweden; 4 Department of Veterinary and Animal Sciences, University of Copenhagen, Copenhagen, Denmark; 5 Department of Laboratory Medicine, Medical School, University of Pécs, Pécs, Hungary; 6 Department of Biophysics, Medical School, University of Pécs, Pécs, Hungary; McMaster University, Hamilton, Ontario, Canada

**Keywords:** drug resistance evolution, drug resistance mechanisms, ionophore, purine metabolism, fitness, cross-resistance, mouse experiment

## Abstract

Ionophores are antibacterial compounds that affect bacterial growth by changing intracellular concentrations of the essential cations, sodium and potassium. They are extensively used in animal husbandry to increase productivity and reduce infectious diseases, but our understanding of the potential for and effects of resistance development to ionophores is poorly known. Thus, given their widespread global usage, it is important to determine the potential negative consequences of ionophore use on human and animal health. In this study, we demonstrate that exposure to the ionophore monensin can select for resistant mutants in the human and animal pathogen *Staphylococcus aureus*, with a majority of the resistant mutants showing increased growth rates *in vitro* and/or in mice. Whole-genome sequencing and proteomic analysis of the resistant mutants show that the resistance phenotype is associated with de-repression of *de novo* purine synthesis, which could be achieved through mutations in different transcriptional regulators including mutations in the gene *purR*, the repressor of the purine *de novo* synthesis pathway. This study shows that mutants with reduced susceptibility to the ionophore monensin can be readily selected and highlights an unexplored link between ionophore resistance, purine metabolism, and fitness in pathogenic bacteria.

**IMPORTANCE:**

This study demonstrates a novel link between ionophore resistance, purine metabolism, and virulence/fitness in the key human and animal pathogen *Staphylococcus aureus*. The results show that mutants with reduced susceptibility to the commonly used ionophore monensin can be readily selected and that the reduced susceptibility observed is associated with an increased expression of the *de novo* purine synthesis pathway. This study increases our understanding of the impact of the use of animal feed additives on both human and veterinary medicine.

## INTRODUCTION

Ionophores are chemicals that are an important part of feed given to ruminant animals and poultry (1–3) to improve feed efficiency and prevent the parasitic disease coccidiosis. The effects of ionophores in ruminants are partly attributed to their ability to alter the animal’s intestinal microbiome composition (4, 5). For example, one of the most used ionophores, the polyether monensin, exerts its effects by altering the rumen’s microbial populations, resulting in a reduction in volatile fatty acid synthesis and amino acid degradation, thereby reducing the loss of carbon and nitrogen sources (6–8). The resulting increase in energy availability and nitrogen retention improves the efficiency of feed utilization and the productivity of the ruminant animal (4, 9). Monensin treatment also reduces morbidity and mortality by reducing the growth of bacteria that cause ruminal acidosis, bloating, and bovine emphysema, respectively (1, 10, 11). Furthermore, monensin has a coccidiostatic effect and is widely used to prevent/treat coccidiosis, mainly in poultry (12, 13). Improved growth characteristics and reduction in disease in production animals have been shown to translate into substantial economic gains in these industries, making the use of ionophore additives a very appealing practice (14, 15).

The ability of ionophores to alter microbial species composition stems from the fact that these lipid-soluble compounds can reversibly bind ions and transport them across a hydrophobic cell membrane (16). This property allows them to affect the intracellular concentrations of biologically important cations, particularly in Gram-positive bacteria, which are generally more susceptible to ionophores than Gram-negatives (17). Monensin, for example, binds to sodium and potassium ions transporting them either into the cell or outside the cell, respectively (16, 18), with this exchange also requiring an equivalent transport of hydrogen ions. Subsequently, these changes in intracellular ion concentrations result in multiple downstream effects, including disruption of the proton motive force (affecting nutrient transport and ATP synthesis), and a reduction in intracellular pH (19).

In spite of the widespread use of ionophores and a substantial understanding of their chemical properties, our understanding of the emergence and mechanisms of ionophore resistance, as well as its implications for other relevant phenotypes in bacteria, is very limited. Existing studies on ionophore resistance suggest that changes in the cell membrane, expression of efflux pumps, and increased synthesis of extracellular polysaccharides may contribute to ionophore resistance (20, 21). Despite these studies, it is still unclear if the development of resistance is due to stable genetic mutations or unstable reversible physiological changes (22, 23). These are important questions to investigate since resistance development to ionophores could result in potentially negative consequences for human and animal health. Importantly, the use of ionophores could result in the selection of ionophore resistance in important human pathogens, like *Staphylococcus aureus*, that are often present in ruminant animals. How these changes might affect the pathogen’s cross-resistance to medically relevant antibiotics and its virulence is unclear. To the best of our knowledge, only one study has examined whether ionophore resistance mechanisms can provide cross-resistance to antibiotics in relevant animal and human pathogens (24). Results from this study suggest that there is no cross-resistance to antibiotics in ionophore-resistant mutants of *Clostridium aminophilum* F.

Here, we present evidence that ionophore resistance can emerge in the human pathogen *S. aureus* through upregulation of the *de novo* purine synthesis pathway. *De novo* purine synthesis is an important metabolic pathway where the metabolites contribute to the synthesis of important biomolecules and in cell signaling, and play a role in extracellular matrix formation (25, 26). Besides these functions, this pathway has also been linked to increased levels of virulence and enhanced biofilm formation in human pathogens (26–29). In line with these observations, we demonstrate that ionophore-resistant *S. aureus* mutants display increased virulence as well as faster growth *in vitro*. Our study thus demonstrates an important and unexplored link between ionophore resistance, purine biosynthesis, and fitness in these significant human and animal pathogens.

## RESULTS

### Mutations in genes coding for proteins involved in the purine biosynthesis pathway and Na^+^/H^+^ antiporter systems generate monensin resistance

Monensin-resistant *S. aureus* mutants were selected by plating an overnight-grown culture of susceptible *S. aureus* at four different concentrations of monensin: 0.5, 1, 2, and 4 µg/mL. The experiment was done as a fluctuation assay, and resistant mutants were observed at all the concentrations tested, with resistance mutation rates varying between 1 × 10^−10^ and 1 × 10^−9^ per cell per generation at these concentrations. Five resistant mutants that were selected at 4 µg/mL were then chosen for further characterization. The minimum inhibitory concentrations (MICs) for all these resistant mutants were approximately twofold higher than the susceptible *S. aureus* (MIC_susceptible_ = 2–4 µg/mL; Table 1), as determined by broth microdilution (BMD). Time-kill experiments were performed to further characterize the reduced susceptibility of these mutants to monensin (Fig. S1). Thus, the growth of the susceptible and resistant *S. aureus* strains was measured at four different monensin concentrations (0, 4, 16, and 64 µg/mL) for a period of 20 h. The susceptible *S. aureus* grew only in the absence of monensin, whereas in the presence of monensin, the colony-forming units (CFUs) decreased by approximately 2 logs over 20 h. In contrast, all the resistant mutants grew at concentrations of 4 µg/mL and above. Interestingly, for four resistant mutants, we observed a stronger inhibitory effect of 16 µg/mL of monensin compared to 64 µg/mL. More work is needed to determine the reason behind this observation.

**TABLE 1:** Characterization of monensin-resistant mutants used in the study^*a*^

Strain	Genotype	MIC µg/mL	Relative exponential growth rate (with respect to the susceptible *S. aureus*)
DA28823 (USA300 JE2)	–	2–4	–
DA65169	*rpoB* P963S *pknB* N277fs *SAUSA300-0137* Q186*	4–8	1.03 ± 0.08
DA65173	*rsbU* 245del *apt* F74Y *mnhF* L19dup	4–8	1.32 ± 0.14
DA65174	*apt* I159fs *rpoE* P93fs	4–8	1.11 ± 0.02
DA65175	*mnhA* G402S *rpoF* N73fs	4–8	1.20 ± 0.06
DA65177	*purR* K120fs	4–8	1.22 ± 0.09
DA66382	*purR*::Tn	4–8	1.20 ± 0.03

^*a*^ Mutations identified by whole-genome sequencing and the MICs for different monensin-resistant mutants.

**Fig 1:**
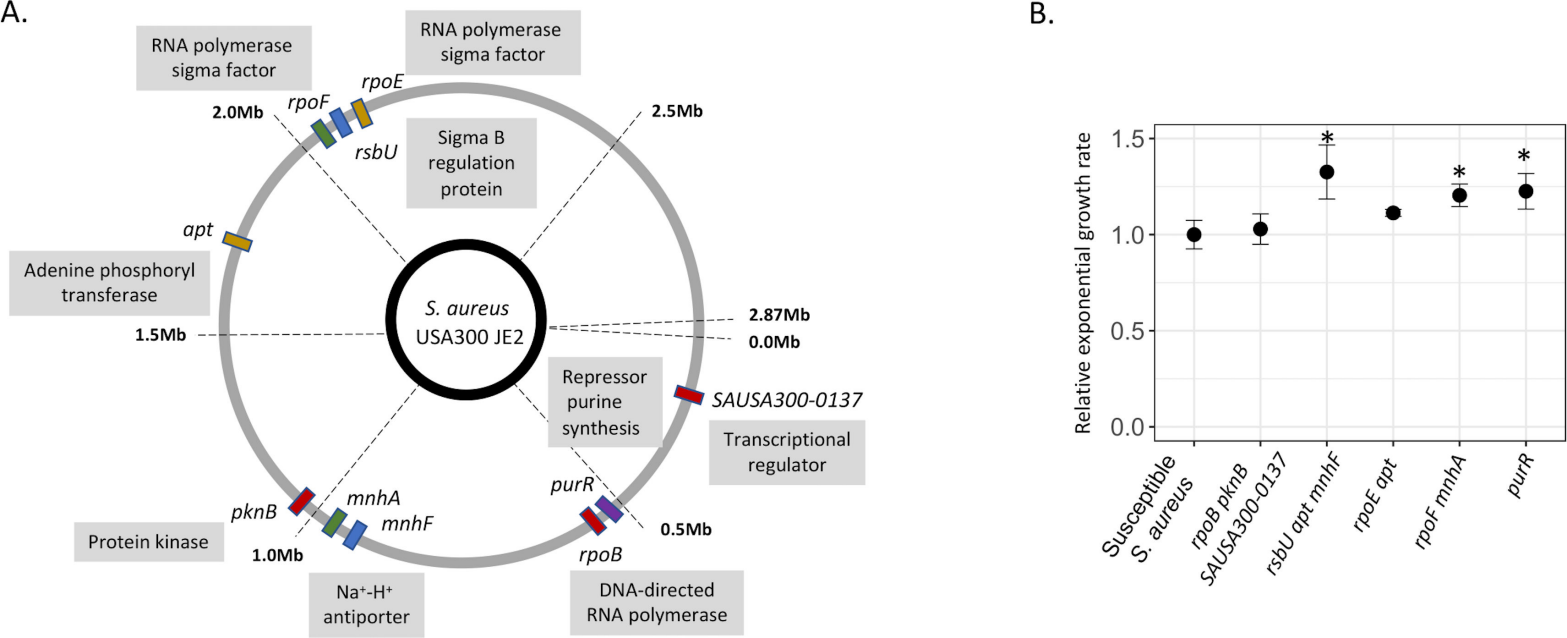
Characterization of monensin-resistant mutants. (**A**) Whole-genome sequencing analysis was performed for five monensin-resistant mutants. Mutations observed within a single mutant are depicted with the same color. (**B**) Relative exponential growth rate of the resistant mutants was measured relative to the susceptible *S. aureus* (growth rate set to 1.0). Four replicates were used in each case, and error bars represent standard deviation. Statistical significance was determined by performing a one-way ANOVA followed by a Tukey HSD test and is denoted as *.

To identify which mutations conferred monensin resistance, the susceptible parental strain and five resistant mutants were whole-genome sequenced. All but one mutant contained more than one mutation (Fig. 1A), with 11 different mutations observed across the five mutants. The only gene that had mutations in two independent mutants was the *apt*, which encodes the adenine phosphoribosyl enzyme and is involved in the purine biosynthesis pathway. Two other mutants also harbored mutations in genes that encode regulators of the purine biosynthesis pathway: the *purR* gene, a repressor of purine biosynthesis pathway, and the *pknB* gene encoding a serine/threonine kinase that affects the expression of genes involved in the purine and pyrimidine biosynthesis pathway, in the cell wall metabolism, and in the glutamine synthesis pathway (30). The resistant mutant with the *purR* mutation was the only mutant where only a single mutation was observed to be responsible for the resistance phenotype. Besides these mutations that targeted the purine biosynthesis pathway, mutations were also observed in genes encoding for transcriptional factors or regulators of transcriptional factors; these included mutations in genes *rpoB* (DNA-directed RNA polymerase), *SAUSA300-0137* (uncharacterized transcriptional regulator), *rpoE* (DNA-directed RNA polymerase), *rpoF* (DNA-directed RNA polymerase), and *rsbU* (Sigma-B regulatory protein). Mutations were also observed in the genes encoding for Na^+^/H^+^ antiporter systems *mnhA* and *mnhF* (Fig. 1A).

To determine the fitness costs of the resistance, the relative exponential growth rate of all five mutants was compared to the susceptible parental *S. aureus*. Unexpectedly and importantly, three out of five mutants showed a substantial (10%–30%) increase in relative growth rate as compared to the susceptible *S. aureus*, while the growth rates of the other two mutants were similar to that of the susceptible *S. aureus* (one-way ANOVA, *F* value = 8.499, and *P* = 9.33E-05, followed by Tukey honestly significant difference (HSD) for individual comparisons; Fig. 1B).

### Monensin-induced increases in intracellular Na^+^:K^+^ ratios are dampened in resistant *S. aureus*

Monensin has been shown to affect intracellular ion concentrations in bacteria that could, either by itself or through downstream effects (e.g., by alterations in pH, ATP:ADP ratios), lead to growth inhibition (31, 32). Thus, it is possible that the resistance mutations may confer their effect by counteracting monensin-induced alterations in ion concentrations. To determine if monensin affected the resistant and susceptible *S. aureus* strains differentially, we measured intracellular concentrations of Na^+^:K^+^, intracellular pH, and intracellular levels of ATP:ADP in four different resistant mutants and the susceptible *S. aureus*. In each case, the strain was grown to mid-log phase (OD_600_ = 0.4) and then treated with monensin for either 1 h (for intracellular ATP:ADP) or 5 h (for intracellular ion concentration and intracellular pH measurements), after which point the assay was performed.

**Fig 2:**
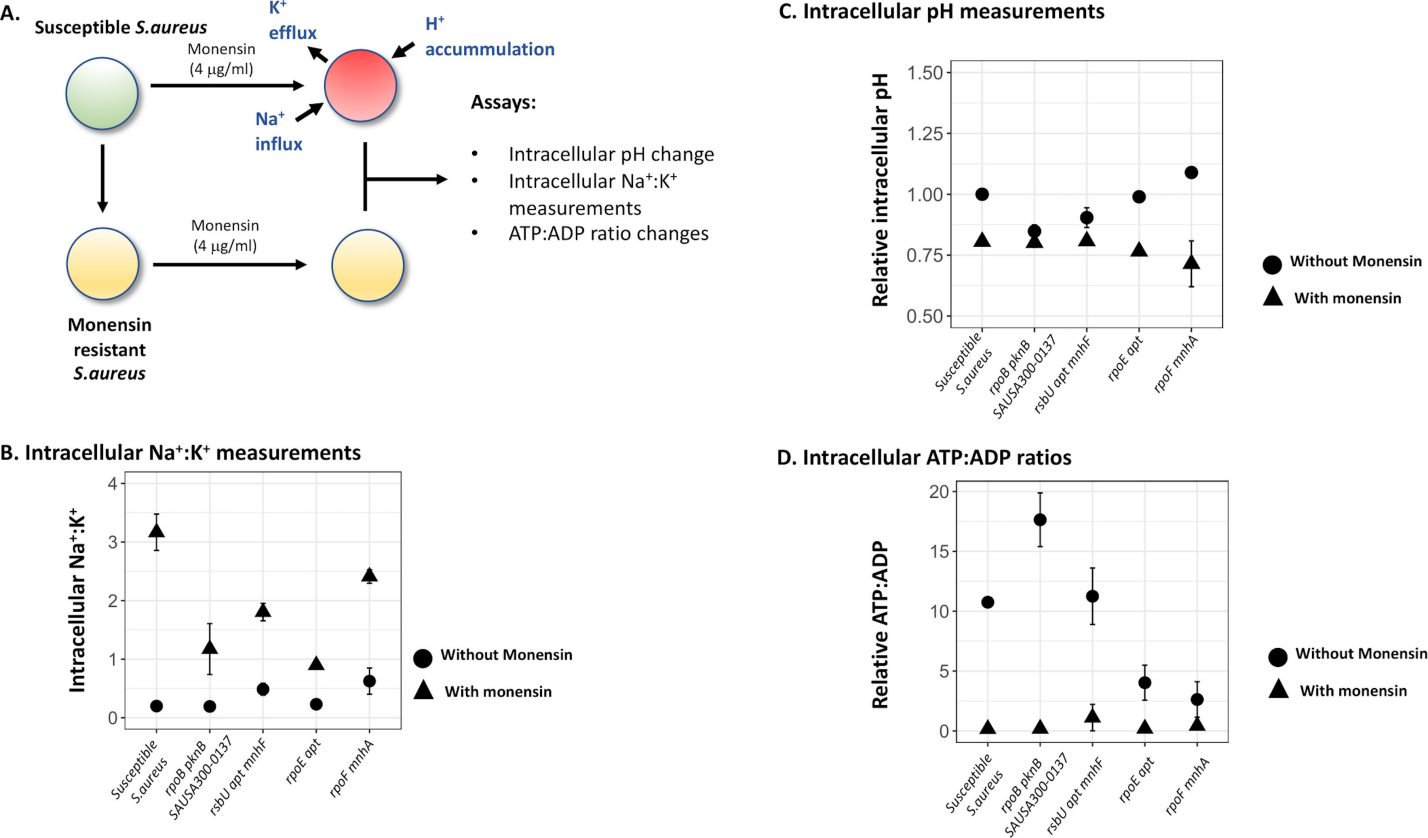
Monensin-induced physiological changes in the susceptible and resistant *S. aureus* strain. (**A**) Known effects of monensin on the intracellular ion concentrations are shown for susceptible *S. aureus* strain, which includes efflux of K^+^ ions and an influx of Na^+^ and H^+^ ions. These changes were experimentally determined for the monensin-resistant and monensin-susceptible *S. aureus*: (**B**) changes in intracellular pH, (**C**) intracellular Na^+^:K^+^ ratio, and (**D**) intracellular ATP:ADP ratio were determined for the susceptible and four monensin-resistant *S. aureus* strains. In each case, circles denote measurements in the absence of monensin, and triangles show measurements in the presence of monensin. Two replicates are used in each case, and error bars represent standard deviation.

For all five strains tested, we observed, as expected, a substantial increase in the ratio of intracellular Na^+^:K^+^ during exposure to monensin; however, this increase was different between the susceptible (~16-fold) and monensin-resistant mutants (approximately four- to sixfold; two-way ANOVA, *F*_gen. × mon. treatment_ = 11.955, *P* = 0.00324). Monensin exposure also caused a reduction in the intracellular pH in the susceptible and two of the resistant *S. aureus* strains (Fig. 2C, two-way ANOVA, *F*_mon. treatment_ = 19.37, *P* = 0.0004); similar results were observed for intracellular ATP:ADP ratios, where monensin treatment resulted in a reduction in the intracellular ATP:ADP ratios in the susceptible and two resistant *S. aureus* strains (two-way ANOVA, *F*_mon. treatment_ = 17.607, *P* = 0.0006, Fig. 2D). Interestingly, there was no difference observed in measurements of intracellular pH (two-way ANOVA, *F*_gen. × mon. treatment_ = 0.001, and *P* = 0.97; Fig. 2C) and ATP:ADP ratios (two-way ANOVA, *F*_gen. × mon. treatment_ = 0.086, and *P* = 0.77; Fig. 2D), between the susceptible and resistant *S. aureus* strains due to monensin exposure. Overall, these results show that the only response common for the four resistant mutants was a dampening of the monensin-induced increase in Na^+^:K^+^ ratio, but for intracellular pH or ATP:ADP ratios without or with monensin present, no common change was observed in the mutants.

### The purine biosynthesis pathway is upregulated in all the resistant mutants

**Fig 3:**
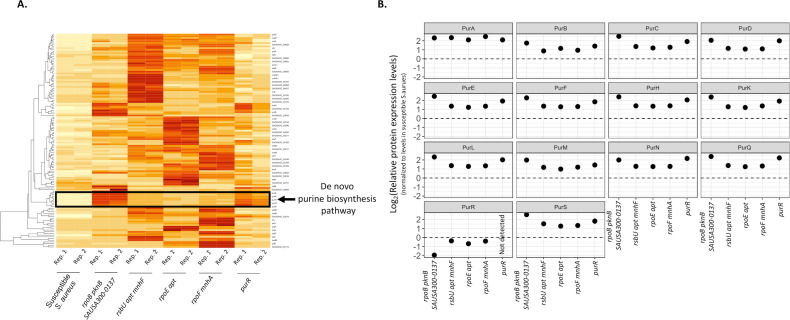
Whole-cell proteomic analysis of monensin-resistant mutants. (**A**) A heatmap depicting a subset of proteins with an increased level of protein expression in monensin-resistant mutants, as compared to the susceptible *S. aureus*. Darker colors represent a higher level of expression. Proteins involved in the *de novo* purine synthesis pathway are highlighted. Two replicates were used in each case. (**B**) Expression levels of the different proteins involved in the *de novo* purine synthesis pathway in the monensin-resistant mutants, relative to the susceptible *S. aureus*. Expression levels are plotted on a log_2_ scale.

Since several of the monensin-resistant mutants that were described above had mutations in general transcriptional regulators, whole-cell proteomic analysis was performed to identify how these mutations were affecting the expression of proteins involved in different cellular processes that could potentially contribute to ionophore resistance. This analysis was performed for the susceptible and five different resistant *S. aureus* strains in the absence of monensin exposure. Across all five mutants, as compared to the susceptible *S. aureus*, 128 proteins were upregulated and 317 proteins were downregulated (a difference of twofold or greater in each case; Table S1). An analysis of the upregulated proteins among the resistant mutants showed significant enrichment of proteins involved in the purine biosynthesis pathway (Fig. 3) and the alpha-amino acid synthesis. Furthermore, all the resistant mutants also showed a downregulation of the PurR repressor protein (Fig. 3B), which is in accordance with the upregulation of proteins involved in the purine biosynthesis pathway in these mutants.

**Fig 4:**
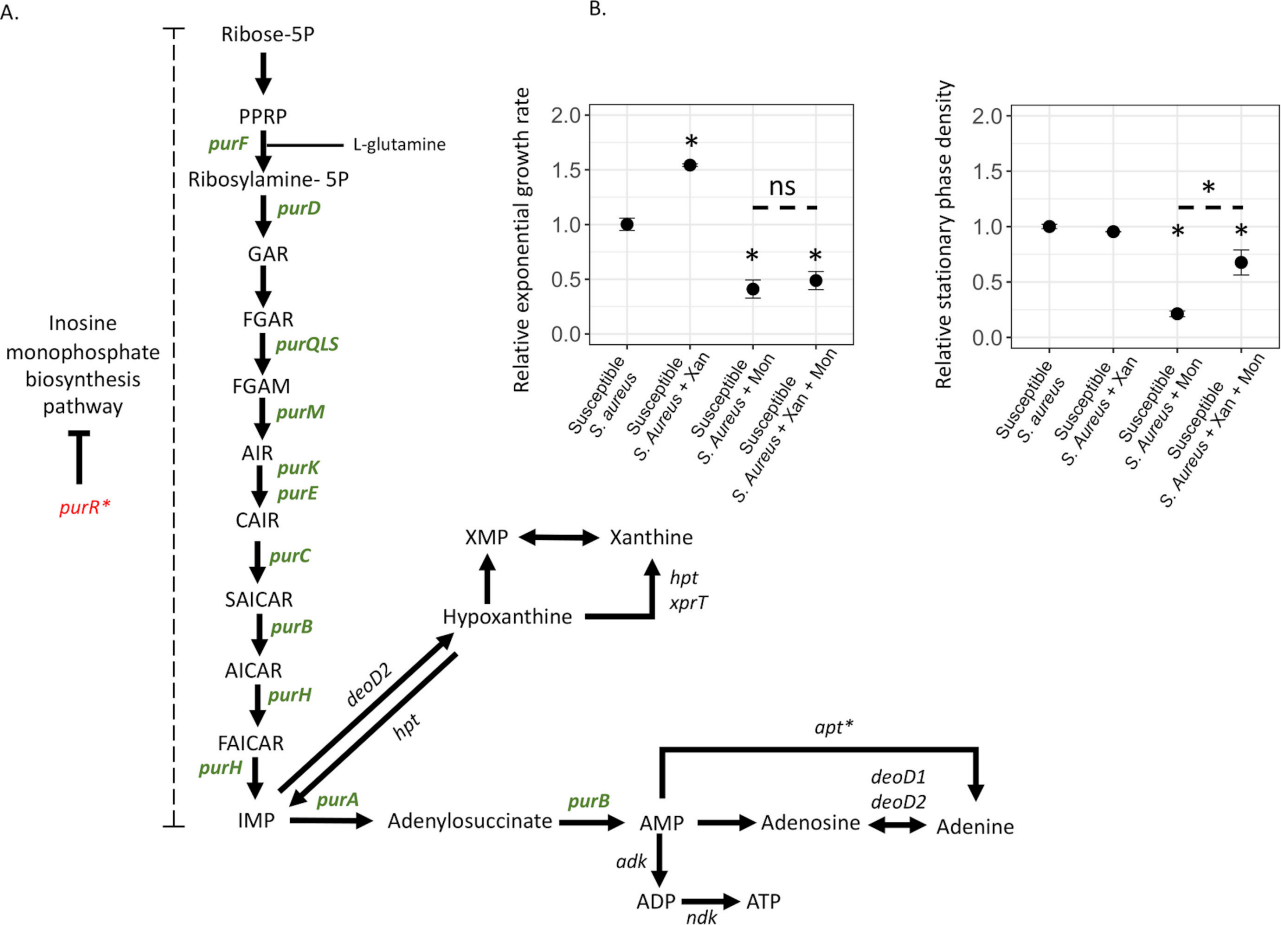
Upregulation of *de novo* purine biosynthesis pathway observed in monensin-resistant mutants. (**A**) The *de novo* purine synthesis pathway is depicted with proteins that were upregulated in the monensin-resistant mutants shown in green and those that were downregulated shown in red, both relative to the susceptible *S. aureus*. (**B**) The effect of monensin on the susceptible *S. aureus* is reduced in the presence of xanthine, a metabolite of the purine *de novo* synthesis pathway. Exponential growth rate and stationary phase densities are plotted for the susceptible *S. aureus* in the absence of xanthine and monensin, in the presence of xanthine, in the presence of monensin, and in the presence of xanthine and monensin together. Four replicates were used in each case, and error bars represent standard deviation. Statistical significance was determined by performing a two-sided Student’s *t*-test at *P* < 0.05 and is denoted as *, while non-significance is shown as ns.

The sequencing and proteomic analysis showed that increased *de novo* purine synthesis was a common denominator in the resistant mutants and therefore was potentially involved in conferring monensin resistance. We reasoned that if an increase in purine levels can confer resistance, this implies that the inhibitory effect of monensin on a susceptible bacterium can be counteracted by providing purine precursors. To this end, we determined if susceptible wild-type *S. aureus* became more resistant toward monensin in the presence of xanthine, a key metabolite of the purine synthesis pathway (Fig. 4A). First, we determined the effect of xanthine on the MIC of monensin for the susceptible *S. aureus* using a BMD assay. Although no change in the MIC for the susceptible *S. aureus* was observed, we did observe improved growth at lower concentrations of monensin in the presence of xanthine. To confirm this observation, we analyzed the growth curves of the susceptible *S. aureus* strain at the sub-MIC of monensin (2 µg/mL), in the presence and absence of xanthine. For each condition, we determined the exponential growth rate and the stationary phase density (Fig. 4B). In the absence of monensin, xanthine increased the exponential growth rate of the susceptible *S. aureus* by 50% (relative exponential growth rate = 1.54 ± 0.01, Student’s *t*-test *t*_6_ = 19.03, and *P* = 4.08E-06, corrected for multiple testing using Bonferroni’s correction) but reduced the stationary phase density by 5% (relative stationary phase density = 0.95 ± 0.001, Student’s *t*-test *t*_6_ = 4.61, and *P* = 0.014, corrected for multiple testing using Bonferroni’s correction). In the presence of monensin, xanthine did not affect the exponential growth rate of susceptible *S. aureus*, and the relative exponential growth rate in the presence of monensin (as compared to its absence) was 0.41 ± 0.08, while in the presence of monensin and xanthine, it was 0.49 ± 0.08 (Student’s *t*-test *t*_6_ = 1.33 and *P* = 0.69, corrected for multiple testing using Bonferroni’s correction). Most importantly, xanthine counteracted the inhibitory effect of monensin in the stationary phase of susceptible bacteria. Thus, monensin alone strongly reduced the stationary phase density (relative stationary phase density = 0.21 ± 0.02), but this inhibition was abrogated in the presence of xanthine (relative stationary phase density = 0.67 ± 0.12, Student’s *t*-test *t*_6_ = 7.56, and *P* = 0.0012, corrected for multiple testing using Bonferroni’s correction). In summary, these results indicate that increased purine levels, generated either by de-repressing mutations or by the addition of purine metabolites, can generate monensin resistance.

### Monensin-resistant *S. aureus* displays increased virulence

**Fig 5:**
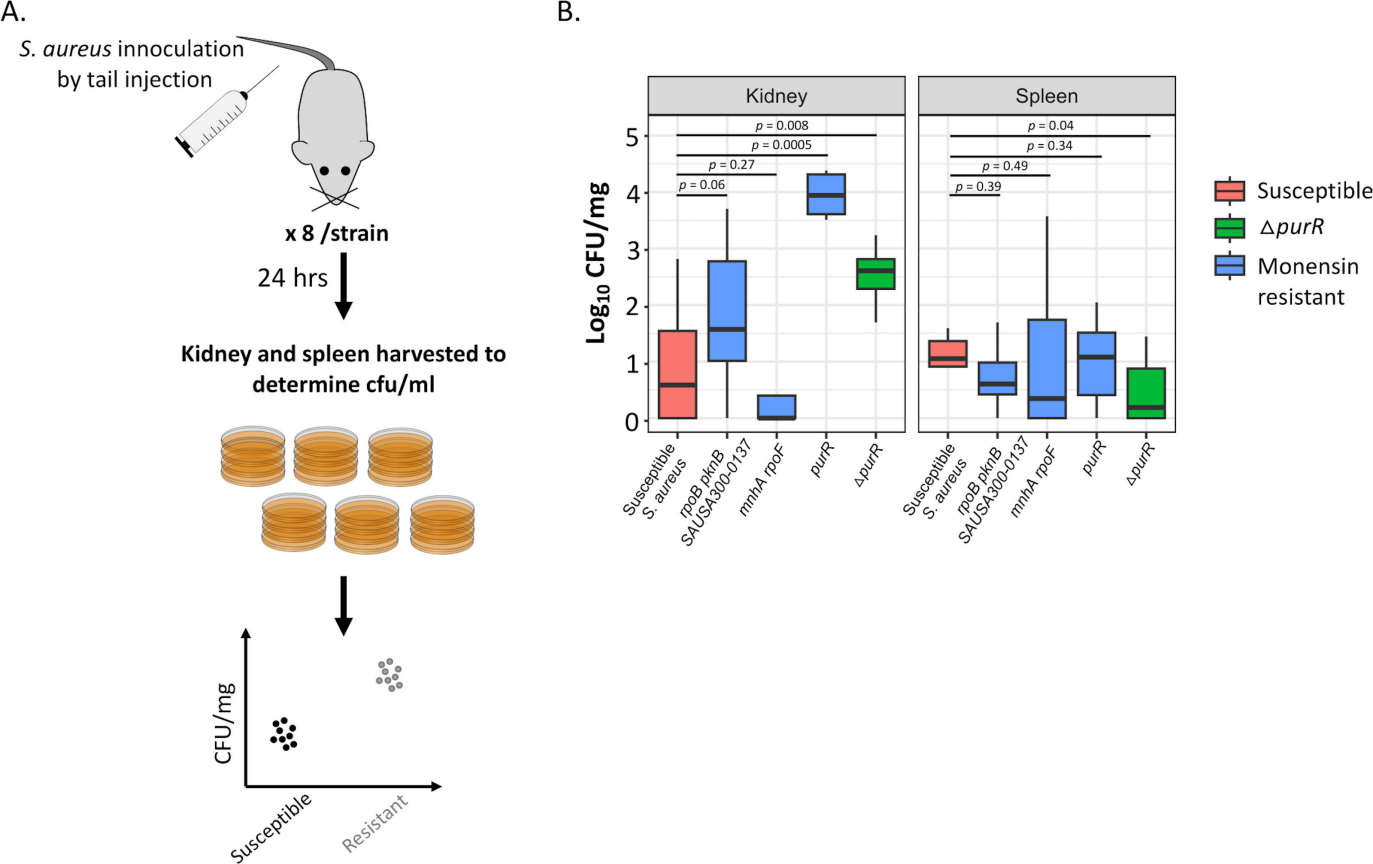
Animal infection experiments to determine the bacterial load of monensin-resistant mutants. (**A**) Experimental design depicting the infection experiment in mice using monensin-susceptible and monensin-resistant *S. aureus*. Eight mice were infected by tail injection for each strain. The kidney and spleen from each mouse were then harvested 24 h post-infection, and the bacterial load was determined by plating on TSA plates. (**B**) Bacterial load in the kidney and spleen are shown for the susceptible (red) and three monensin-resistant (blue) *S. aureus* strains. A Δ*purR S. aureus* mutant (green) was used as a positive control in the experiment. Eight replicates are used in each case, and *P*-values are calculated for a two-sided Student’s *t*-test.

Previous studies showed that upregulation of the *de novo* purine synthesis pathway results in increased virulence of *S. aureus* (28, 33, 34). To determine if the monensin-resistant mutants observed in our study that displayed an upregulated *de novo* purine synthesis pathway also demonstrated increased virulence as measured by growth in mice, we performed experimental infection of mice with the susceptible and resistant *S. aureus* strains. Thus, eight mice were each injected with five different *S. aureus* strains (one susceptible, four monensin-resistant mutants, three of which were isolated in our selection, and one constructed *purR* insertion mutant), and 24 h of post-infection, the kidney and spleen of the mice were harvested to measure for the levels of systemic growth (Fig. 5). As compared to the susceptible *S. aureus*, we observed a higher bacterial load (1 to 3.5 logs) for three monensin-resistant *S. aureus* strains in the kidney, but not in the spleen. Among these, the first mutant (DA65169) had mutations in the genes *rpoB* (DNA-directed RNA polymerase), *pknB* (protein kinase), and SAUSA300-0137 (transcriptional regulator), while the second mutant (DA65177) had an insertion in the gene *purR*. Both of these mutants also displayed the largest decrease in the expression of the PurR protein (Fig. 3B). Thus, the relative expression of the *purR* gene with respect to the susceptible ancestral *S. aureus* was −1.94 ± 0.03 on a log_2_ scale for DA65169, while this protein was not detected at all in DA65177. The third monensin-resistant *S. aureus* strain (DA65175) did not show any difference as compared to the susceptible *S. aureus* in both organs. The relative expression of the PurR protein in this mutant was not as low as that in the other mutants (−0.40 ± 0.05 on a log_2_ scale), which might explain the absence of an effect in the mice. These results demonstrate that the lowered expression of the PurR protein plays a key role in conferring monensin resistance, while also contributing to *S. aureus* becoming more virulent, as demonstrated by increased growth in kidneys. Why this growth-enhancing effect was not observed in the spleen is at present unclear.

### Cross-resistance to antibiotics and other ionophores

To determine if the mutations that confer monensin resistance can also increase resistance toward other antibacterial agents; MIC for the monensin-resistant mutants was measured for other ionophores (salinomycin, narasin, and lasalocid A), antibiotics (gentamicin, tetracycline, vancomycin, daptomycin, polymyxin B, colistin, and neomycin), and antimicrobial peptides (protamine and gramicidin). Monensin-resistant mutants did not show any change in resistance levels to the other ionophores tested in our study (Table S2). For the antibiotics and antimicrobial peptides, the monensin-resistant mutants displayed increased susceptibility to daptomycin, polymyxin B, colistin, and protamine, although to varying degrees. Thus, one of the resistant mutants (DA65169) showed increased susceptibility to protamine, and two mutants (DA65173 and DA65175) showed increased susceptibility to daptomycin, while three resistant mutants (DA65173, DA65174, and DA65175) showed increased susceptibility to polymyxin B and colistin.

## DISCUSSION

In summary, this study shows that it is possible to select *S. aureus* mutants with reduced susceptibility to the feed-additive monensin. Mutants emerged with a rate of 10^−10^ to 10^−9^ per cell per generation (similar to the mutation rates for some antibiotics), and they showed significant changes in important phenotypes. Thus, the mutants displayed significantly increased growth rates *in vitro* as well as increased growth in a mouse model, implying that, even in the absence of selection, they could be maintained in a population (35) and potentially also be causing more virulent infections by virtue of increased bacterial loads. The mutants did not show cross-resistance to antibiotics or antimicrobial peptides, but they were more susceptible to the antibiotics polymyxin B and colistin and the antimicrobial peptide protamine. The genetic analysis of these mutants demonstrated that mutations in several different genes, including transcriptional regulators and ion transporters, contributed to monensin resistance. A few previous studies have investigated the emergence of ionophore resistance, but they have lacked either an analysis of the underlying genetic/physiological mechanism(s) or a description of key bacterial phenotypes (growth, virulence, and cross-resistance). Some studies have investigated resistance to monensin and suggested that thickening of the cell wall in different *Enterococcus* species (23) and increased extracellular polysaccharide in *C. aminophilum* F^21^ were the underlying causes of resistance; however, in both of these studies, the genetic mechanisms for resistance were unknown. Other studies have investigated the resistance to the ionophores tetronasin and narasin in *Streptomyces longisporoflavus* and *Enterococcus faecium*, respectively, and shown that upregulation of ABC-type efflux pumps that reduced the intracellular concentrations of the ionophores was the likely cause of resistance (20, 36, 37). *E. faecium* efflux pump mutants were also shown to be resistant to ionophores, salinomycin and maduramicin, but not to monensin or to other clinically relevant antibiotics (20).

An important question is how the different mutations that we observed in our study act to confer resistance. Since it is still unclear what the ultimate cause of ionophore-induced growth inhibition is, it is also difficult to explain how the resistance mutations prevent inhibition, in particular since many of the mutations are in transcriptional regulators (RsbU, RpoE, RpoF, and PurR) that cause global pleiotropic effects. Previous study (19) and this work have indeed shown that the Na^+^:K^+^ ratio is increased by monensin, but whether this is the direct cause of inhibition or whether one or several downstream effects (altered pH, ATP:ADP ratios, changed proton motive force, altered gene regulation, metabolism, etc.) are also contributing remains unclear. Our analysis of the resistant mutants suggests that they share at least two physiological responses (in spite of their different mutations) that are likely to confer increased resistance: (i) a dampening of the monensin-induced increase in Na^+^:K^+^ ratio and (ii) an upregulation of purine synthesis. Whether these two effects are independent of each other or physiologically linked is at present unclear, but it is interesting to note that *purR* inactivation alone (resulting in upregulation of purine biosynthesis) is sufficient to confer resistance.

A particularly important finding of this study is the new link between ionophores and purine metabolism. Purines are integral components of several cellular macromolecules and play a significant role in energy metabolism and cell signaling in bacteria (25, 26, 38). Their importance to bacterial physiology has also resulted in pathways involved in purine metabolism being important targets for antibiotic development (38). The results presented in this study add to the significance of these pathways by associating them with ionophore resistance. Thus, we observed an upregulation in *de novo* purine synthesis among all the monensin-resistant mutants characterized in this study. Whole-genome sequencing analysis of the mutants revealed that this could be achieved through different sets of mutations. Thus, we observed mutations in several transcriptional regulators including *rpoB* (DNA-directed RNA polymerase), *rpoE* (DNA-directed RNA polymerase), *rpoF* (DNA-directed RNA polymerase), *rsbU* (Sigma-B regulation protein), and *purR* (repressor of purine biosynthesis pathway). A link between purine metabolism and monensin resistance was further demonstrated by xanthine-feeding experiments, where the susceptible *S. aureus* displayed better growth at sub-MIC of monensin. Although the central role of purines in cellular physiology makes it difficult to assess if the bactericidal effect of ionophores is either due to purine starvation or alternatively due to a purine-dependent phenotype, the results presented in this study do link purine metabolism to monensin resistance.

Upregulation of the *de novo* purine synthesis pathway has been shown to contribute to the virulence of *S. aureus* through increased synthesis of purine metabolites (33) and due to the regulation of virulence factors by the protein PurR (39), the repressor of the purine synthesis pathway. Thus, *purR* knockout mutants of *S. aureus* have an increased ability for intracellular growth, increased capacity to form biofilms, and increased expression of virulence factors (33, 34, 39). The mouse infection experiments performed in this study demonstrate similarly that the monensin-resistant mutants show an upregulation of the *de novo* purine synthesis pathways, which is associated with an increased virulence (as measured by increased bacterial loads in mice) in three out of four mutants. In summary, these results demonstrate a novel link between ionophore resistance, purine metabolism, and bacterial growth and virulence.

## MATERIALS AND METHODS

### Bacterial culture, media, and selection of monensin-resistant mutants

The susceptible *S. aureus* strain used in our study was *S. aureus* USA300 JE2 (40), which is a derivative of the methicillin-resistant *S. aureus* USA300. It has been cured of two plasmids and is less virulent than the ancestral *S. aureus* USA300. The liquid medium used for the growth of strains and fluctuation assays is the Mueller-Hinton II (MHII) broth (cation-adjusted; Sigma-Aldrich), and the solid medium used is the MHII agar (cation-adjusted; Sigma-Aldrich). Monensin-resistant mutants were isolated by performing a fluctuation assay. Briefly, 30 independent cultures were started using 10^−6^ dilutions of an overnight-grown *S. aureus* culture, such that each culture was started with approximately 1,000 cells. The next day, 100 µL from each culture was plated on MHII plates containing either 0.5, 1, 2, or 4 µg/mL of monensin. All the plates were incubated at 37°C for 3 days, except for the plates containing 4 µg/mL of monensin that was incubated for 6 days. Based on the total number of cells plated, the frequency of mutants at each concentration was calculated, which was then used to calculate the resistance mutation rate using the bz-rates mutation rate calculator (41). The *purR* knockout *S. aureus* (DA66382) used in our studies was obtained from the Nebraska transposon mutant library (40) and is verified to have a single insertion.

### Determination of MIC of monensin for susceptible and resistant *S. aureus* strains

The MIC of monensin for susceptible and resistant *S. aureus* strains was tested by performing a BMD assay and time-kill experiments. For the time-kill experiments, 20 µL of an overnight-grown culture was used to inoculate 2 mL of MHII broth. This was allowed to grow for 1.5 h, after which 20 µL from this culture was used to start each of the four tubes containing 2 mL MHII broth. One hundred microliters from each tube was diluted and plated on MHII plates (observation for *t*_0_). In the remaining volume of each tube, monensin was added to a final concentration of 4, 16, and 64 µg/mL, while no monensin was added in one of the tubes. The cells were allowed to grow, and then CFU/mL was further determined following the same procedure after 2, 4, and 20 h. Two replicates were used in each case.

For the determination of the MIC of monensin for both the susceptible and resistant *S. aureus*, the biological replicates for the same strain showed twofold differences in their respective MICs. In all these cases, additional experiments were performed to determine if this variability could be reduced. However, since this variability was still observed, the MIC values are mentioned as ranges.

### Whole-genome sequencing and analysis

Genomic DNA was extracted using the MasterPure Gram Positive DNA Purification Kit (Epicentre). Instead of the lysozyme delivered with the kit, lysostaphin from *Staphylococcus simulans* (Sigma) (5 mg/mL in 20 mM sodium acetate buffer, pH 4.5) was used. DNA was extracted from 1 mL of overnight-grown culture and was used to make whole-genome DNA paired-end libraries (2 × 300) using Illumina’s Nextera XT kit. These libraries were then sequenced using Illumina’s Miseq platform. Samples were dual-indexed and pooled together. The average whole-genome coverage per sample was 30×, and the sequence data were analyzed using CLC Workbench version 11. In each case, the reads were mapped onto the susceptible *S. aureus* strain (DA28823), which was also whole-genome sequenced.

### Proteomic analysis

Whole-cell proteomics was carried out to get insights into the mechanism of monensin resistance. Overnight-grown cultures of susceptible and resistant *S. aureus* strains were diluted 1:100 in 10 mL MHII broth and were allowed to grow to an optical density (OD_600_) of 0.4. At this point, monensin was added to these cultures at a final concentration of 4 µg/mL. After further letting the cells grow for 1 h, the cells were pelleted down and washed thrice with phosphate-buffered saline (PBS). The cell pellet was then stored at −80°C and was then analyzed by the Proteomics Core Facility at the University of Gothenburg. Briefly, the samples were first lysed, and the total protein concentration in the lysates was measured. An aliquot of each sample was reduced, alkylated, and then digested with trypsin. The digested peptides were then chemically labeled with a tandem mass tag (TMT or TMTpro) reagent. The TMT-based relative quantification was performed largely as described previously (42), with the following modifications: the sample was pre-fractionated via a basic pH reversed-phase high-performance liquid chromatography into 10 fractions, and each fraction was analyzed using the 90-min liquid chromatography-mass spectrometry method. In total, more than 15,000 peptides were identified at a false discovery rate of 1%, making up ~1,900 *S*. *aureus* proteins quantified across the samples.

### Measurement of exponential growth rate and stationary phase density

The growth curves of the susceptible and resistant *S. aureus* strains were measured using a BioscreenC analyzer at OD_600_. Overnight-grown cultures of each strain were diluted 1:1,000 in 1mL MHII broth at the start of the experiment, and optical density was measured every 4 min. These growth curves were then used to determine the exponential growth (using the Kaleidagraph software) and stationary phase density (using R-package growthcurver). Four biological replicates were used in each case.

### Measurements of intracellular Na^+^:K^+^ ratios

The intracellular Na+:K+ ratio of the susceptible and resistant *S. aureus* strains was measured as described elsewhere (43). Briefly, 1 mL of an overnight-grown culture of the appropriate *S. aureus* strain was used to inoculate 1 L of MHII broth. The cells were allowed to grow to an optical density of 0.4, at which point the cells were either harvested or challenged by monensin at a final concentration of 2 µg/mL. The latter of these were then allowed to grow for 5 h, after which the cells were harvested. The harvesting of the cells was performed by centrifuging at 4,500 rpm for 20 min. After the centrifugation, the pelleted cells were transferred to pre-weighed 2 mL Eppendorf tubes and were washed once with distilled water. The washed cells were pelleted again by centrifugation at 13,000 rpm for 5 min. The supernatant was carefully discarded. The cells were centrifuged again for 30 seconds, after which the rest of the supernatant was carefully removed using a pipette. The Eppendorf tube was weighed again to determine the weight of the pellet. The cells were then killed, and the extracellular water was evaporated by heat treatment (100°C for 20 min). The dried pellet was then used for intracellular ion measurements. Briefly, the cell pellets were resuspended in five times the pellet volume of water, frozen, and subsequently boiled to generate a homogenous cell lysate. The slurry was centrifuged for 10 min at 100,000 × *g*. Ion concentrations were measured from the supernatants. Unbound Na^+^ and K^+^ levels were analyzed using a COBAS INTEGRA 400 plus analyzer (Roche Diagnostics, GmbH, Mannheim, Germany) following the manufacturer’s instructions. The intracellular ion concentrations are equal to five times of the measured values.

### Measurement of intracellular pH

The change in intracellular pH of the susceptible and resistant *S. aureus* strains due to the addition of monensin was measured using the BCECF [2′,7′-bis-(2-carboxyethyl)-5-(and-6)-carboxyfluorescein] pH indicator. Two hundred microliters of an overnight-grown culture of the appropriate *S. aureus* strain was used to inoculate 20 mL of MHII broth. The cells were grown to an optical density of 0.4 (600 nm), at which point these were either harvested by centrifugation or treated with 4 µg/mL monensin. The latter were allowed to grow for 5 h after which the cells were harvested by centrifugation. All the pelleted cells were washed thrice with 1 mL Kpi buffer. After the last wash, the cells were pelleted by centrifugation and resuspended in 20 µL of the Kpi buffer. At this point, 1 µL of BCECF pH indicator dye was added to these pelleted cells, followed by a 1-µL addition of 0.5 M HCl. The mixture was vortexed and stored in dark for 10 min. After the incubation, each tube was washed with 1 mL of ice-cold Kpi buffer and was finally resuspended in 2.5 mL of Kpi buffer. The final mixture was then kept at 37°C for 2 min. Three hundred microliters from each tube was then used for fluorescence measurement at 525 and 640 nm over 3 min. The ratio between the fluorescence values at 525–640 nm is taken as a measure of intracellular pH. All the values were then normalized to the values obtained for the susceptible *S. aureus* strain without the addition of monensin. Two replicates were performed in each case.

### Measurement of intracellular ATP:ADP ratios

The change in intracellular ATP:ADP ratio of the susceptible and resistant *S. aureus* strains due to monensin was measured using the ATP/ADP ratio assay kit (Sigma Aldrich, MAK135). Briefly, 50 µL of an overnight-grown *S. aureus* culture was used to inoculate 5 mL of MHII broth. Once the optical density had reached 0.4, the cells were treated with monensin at a final concentration of 4 µg/mL. After 1 h of treatment, 400 µL of cells was centrifuged at 13,000 rpm for 1 min. The supernatant was then discarded, and the pellet was resuspended in a lysis buffer containing 150 µL of Tris-EDTA (10 mM, pH 8), 100 µL of lysozyme (100 mg/mL stock), and 30 µL of proteinase K (20 mg/mL stock). This was followed by three cycles of freezing (−80°C) and thawing (37°C). This mixture was then centrifuged, and 10 µL of the supernatant was then taken for measurements of ATP:ADP ratios, following the instructions from the manufacturers. For all the *S. aureus* strains, the ratios are normalized to values obtained for the susceptible *S. aureus* strain without monensin treatment. Three replicates are used in each case.

### Mouse infection experiments

To determine if monensin resistance affected the levels of virulence in *S. aureus*, mouse infection experiments were performed. Eight 6- to 8-week-old female mice were used for each strain of *S. aureus* tested. The appropriate *S. aureus* strains were grown to an optical density of ~2 (corresponding to ~10^9^ cells/mL) in tryptic soy broth, after which these were centrifuged at 13,000 rpm for 2 min. The supernatant was then discarded, and the pellet was washed with PBS. The washed pellet was then resuspended in 1 mL PBS and then diluted to ~10^8^ CFU/mL. One hundred microliters from this preparation was then used for injecting each mouse in the tail vein. 10^−2^ and 10^−3^ dilutions of this preparation were also plated on tryptic soy agar (TSA) plates to determine the number of cells used for the injection of each mutant. After 24 h of infection, the kidneys and spleen were harvested. Each organ was homogenized, and the undiluted and 10^−1^ and 10^−2^ dilutions were plated on TSA. The plates were incubated for 24 h, after which colonies were counted.

### Determining cross-resistance to other ionophores, antibiotics, and antimicrobial peptides

The MIC of the antibiotics gentamycin, tetracycline, ciprofloxacin, vancomycin, daptomycin, and polymyxin B was determined using Etests, as per the instructions from the manufacturer (bioMerieux). Briefly, overnight-grown cultures of susceptible and resistant *S. aureus* strains were diluted 1:100 and plated using a cotton swab. The Etests were then placed on the plates after which these were incubated at 37°C for 16–18 h. The MIC for other ionophores (salinomycin, narasin, and lasalocid A), for the antibiotic neomycin, and for the antimicrobial peptides (protamine and gramicidin) was determined by performing a BMD assay, using the same protocol as was described earlier for the determination of MIC for monensin.

### Statistical analysis

To determine statistically significant differences, we either used a two-sided Student’s *t*-test or an ANOVA analysis. Thus, a two-sided Student’s *t*-test was performed to determine the effect of xanthine on the growth of the susceptible *S. aureus* at sub-MIC of monensin (Fig. 4B) and to determine the differences in the bacterial load of the susceptible and ionophore-resistant *S. aureus* in the mouse infection experiments (Fig. 5B). Wherever mentioned, Bonferroni’s correction was used to correct multiple comparisons.

A one-way ANOVA was performed to determine statistical differences between the growth rates of susceptible and ionophore-resistant *S. aureus* (different genotypes were considered a factor), followed by Tukey’s HSD test for *post hoc* analysis (Fig. 1B). A two-way ANOVA was performed to determine the differential effect of monensin on intracellular Na^+^:K^+^, intracellular ATP:ADP ratio, and intracellular pH on susceptible and ionophore-resistant *S. aureus* (genotypes and monensin treatment were considered as the two factors; Fig. 2). In the latter case, *F*- and *P*-values were calculated for the effects of each factor as well as the interaction between the factors. Differential effect of monensin on susceptible and ionophore-resistant *S. aureus* was determined based on the *F*- and *P*-values of the interaction term.

## Data Availability

The whole-genome sequence files (fastq) used in this study are deposited at NCBI’s Sequence Read Archive (SRA) under the BioProject ID PRJNA1040000. The mass spectrometry proteomics data have been deposited to the ProteomeXchange Consortium via the PRIDE partner repository with the data set identifier PXD047199 (44).

## SUPPLEMENTAL MATERIAL

Fig. S1 mbio.03155-23-s0001.pdf Time-kill results.

Table S1 mbio.03155-23-s0002.xlsx Proteomics data with unregulated and downregulated proteins.

Table S2 mbio.03155-23-s0003.xlsx Resistance to antibiotics and peptides.

ASM does not own the copyrights to Supplemental Material that may be linked to, or accessed through, an article. The authors have granted ASM a non-exclusive, world-wide license to publish the Supplemental Material files. Please contact the corresponding author directly for reuse.
