# Supplementary material for: Staphylococcus aureus mutants resistant to the feed-additive monensin show increased virulence and altered purine metabolism
Source: mBio. 2024 Jan 12;15(2):e03155-23. doi: 10.1128/mbio.03155-23 (PMC10865815; doi:10.1128/mbio.03155-23)

Supplementary figure 1 : Time-kill data for the susceptible (DA28823) and monensin resistant *S. aureus* strains. Growth of the strains was measured at four different monensin concentrations.

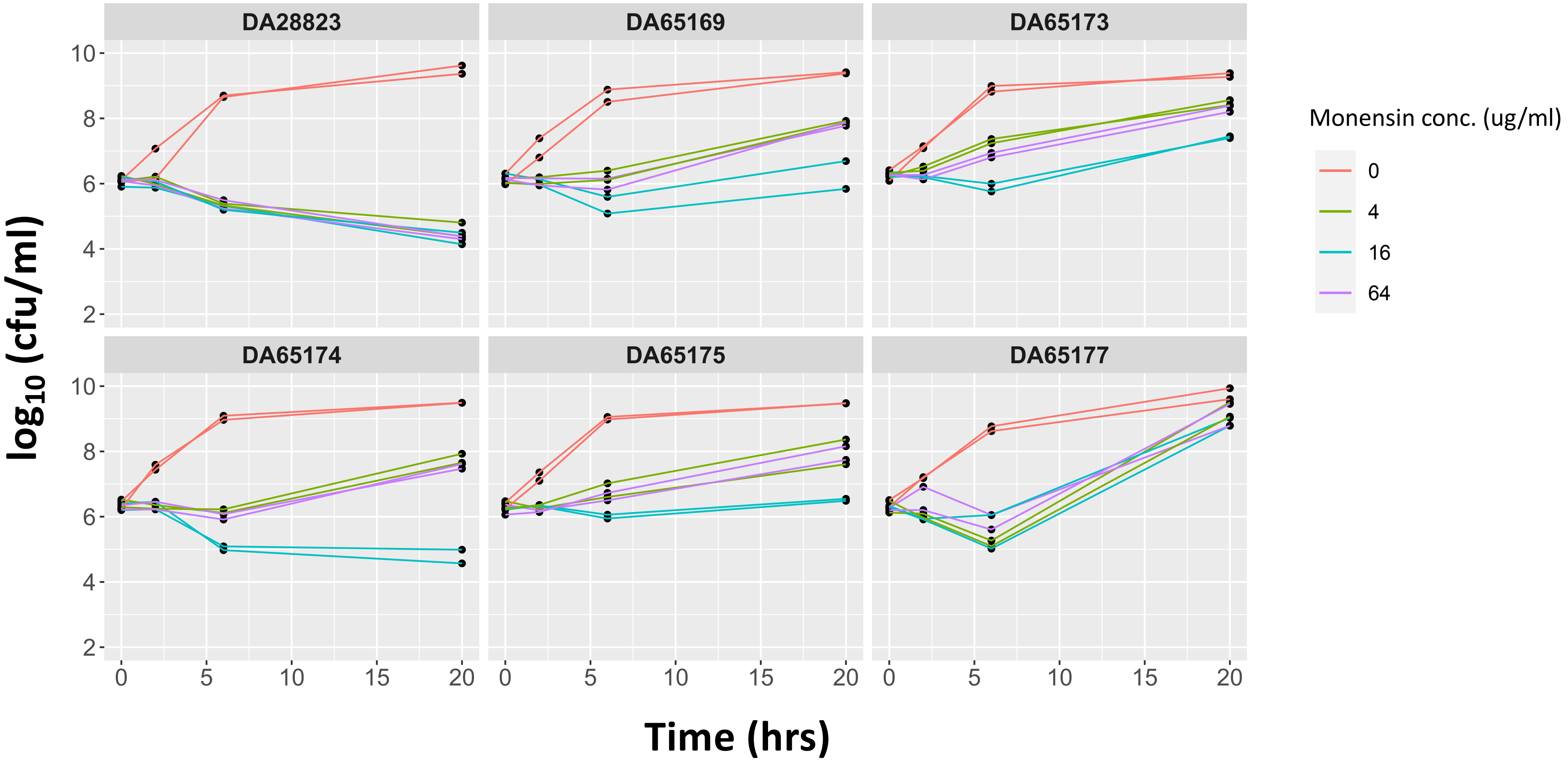

Supplement: Fig. S1 — Time-kill results. [file mbio.03155-23-s0001.pdf]
